# The Papers and Discussions

**Published:** 1911-09-16

**Authors:** 


### THE PAPERS AND DISCUSSIONS.

### A GENERAL SUMMARY.

The meeting just concluded of the British Association will, we believe, rank as one of the most successful ever held, judged from the high order of merit met with in the papers and discussions of the various sections. In point of numbers a small decrease is recorded in the attendance at Portsmouth, but, on the other hand, a larger number than usual of eminent scientists from abroad attended this year's meeting.

Sir William Ramsay's learned and eloquent address has been extensively quoted, and his remarks on the "bribery of scholarships" have excited no little comment, while of the remainder of his speech that portion which sounded a warning note on the subject of our limited supply of coal has aroused the most interest in the public mind.

One of the suggestions made in connection with the concentrating of energy is not without an important bearing on the health of the community. Economical considerations point to the desirability of converting the energy of the coal into electrical energy at the pit-mouth, and this may be associated with the production of half-distilled coal. Now this is a fuel which burns nearly without smoke and is one suitable for domestic grates. It is possible that such a development would prove indirectly a distinct gain, especially in cities, for it seems hardly likely that those systems of domestic heating so common on the Continent and in America will become popular soon enough in this country to make an appreciable abatement of the smoke nuisance.

In the section of Physiology one of the most interesting addresses of the whole meeting was delivered by Professor J. S. Macdonald. In discussing the development of such a perfect physical mechanism as the eyeball, the lecturer pointed out that such a mechanism found external to the central nervous system is capable rather of affecting it than of being affected by it, and this to such a degree that the eye or the ear may be supposed to assist in the development of the central nervous system, instead of the latter assisting in the development of these organs. Then followed the consideration of the part played by external influences in the development of the eye and the ear, and it was suggested that it is probable that orderly electrical forces arrange the developing parts of the eyeball and that natural selection can have done no more than assist in this process. Professor Macdonald incidentally touched upon certain speculations in regard to mind that will doubtless be productive of discussion in many circles. He said there is no scientific evidence either to support or to rebut the statement that the brain is affected by influences other than those which reach it by the definite afferent paths from sense organs. It is possible that the brain is an instrument traversed by an unknown influence which finds resonance within it. An instrument shaped in the embryo by a certain set of conditions may in due course of time become the play of some new influence which have taken no part in fashioning it. In this connection Professor Macdonald found it difficult to refrain from using the word "soul."

In this same section the subject of anæsthetics provided some interesting papers, most of which were in connection with the presentation of the third interim report of the Committee dealing with this subject, and consisting of Dr. A. D. Waller, Sir F. Hewitt, Dr. Blumfeld, Dr. Buckmaster, and Mr. J. A. Gardner.

In the report presented by Dr. Waller it was stated that the laboratory experiments have been confirmed as regards the adequacy of a ten per cent. mixture of ether and air for producing and maintaining a condition of surgical anæsthesia in man; and that in the "open" method of ether administration the percentage of ether delivered is approximately ten per cent. Observations were made to ascertain the maximum percentage afforded to the interior of a face-piece freely supplied with liquid ether by the "open" method, and it was found that this is about twenty per cent. and that it falls after various intervals to between nine and ten per cent. An attempt to raise the percentage to over twelve by excess of ether did not succeed, *i.e.* there is no liability to danger by accidentally exceeding the natural maximum percentage.

Sir Frederic Hewitt admitted his conversion to the "open" as opposed to the "close" method of etherisation, and thought that a new era in anæsthesia was opening in this country. Elaborations and complications have been thrown off one by one till now the procedure was as simple as it can well be; and although appliances for the percentage administration of chloroform had taught them that two per cent. was a safe limit for this anæsthetic, there was every probability that less and less would be heard of chloroform balances, etc., and more and more of this new system of giving ether. Sir Frederic referred also to the reform movement which is demanding legislative control of the administration of anæsthetics, and he hopes that pressure will be put upon the Home Secretary to support the Bill which has been drafted. By a secondary reform of the greatest importance is now pushing its way forward, and that is the replacement of chloroform by ether in general surgery. Even if it were possible to make chloroform sufficiently safe, the complicated apparatus introduced difficulties and objections which in practice are very considerable. He still maintains that a large number of deaths under anæsthetics is preventible, and he is confident that these reforms will materially reduce this number, and he hopes that the Association will support not only the fundamental legislative reform, but also the almost equally important one of encouraging the use of ether by the simple "open" method.

An evening lecture by Dr. Leonard Hill on the "Physiology of Submarine Work" drew together a very large and distinguished audience. The lecture was illustrated by many experiments and served to lay stress on the advantages of the method of decompression by stages over that of slowly and uniformly lowering the atmospheric pressure. It had been shown that decompression on the latter plan might lead first of all to further saturation of the fatty tissues at pressures above three atmospheres; but by rapid decompression to two atmospheres further saturation was prevented. The ideal method included, in addition, oxygen breathing for five minutes and the taking of exercise during the decompression. In the treatment of caisson illness, recompression was the only effective means employed.

In the same section a useful discussion took place on "Ventilation in Confined Quarters," Dr. Leonard Hill opening with a very instructive paper on the physiological and hygienic aspects of the subject. He pointed out that too much attention has been paid to the chemical purity of the air and not enough to the temperature. It has been proved that there is no poisonous organic chemical matter in the products of respiration, and the ill-effects are mainly due to the lack of stimulation by cool air in motion of the temperature regulating mechanism and the cutaneous nerves. The relative humidity of the air is a most important factor and one to which too little attention has been paid; a proper cooling of the body is greatly interfered with when the air is saturated with aqueous vapour, and the whole system suffers in tone, while the amount of energy put out also is greatly diminished. These points have been confirmed by the example of one of the London County Council schools, where the rooms are swept by a gentle current of air at a temperature not above 60° F., and in this school the health and energy of the children are much above the average. Professor Hill entered a plea for the more extended use of the wet-bulb thermometer in workshops, schools, and ships.

Professor Hamburger, of Gröningen, read a paper on the acceleration of phagocytosis by various substances, such as iodoform, chloroform, camphor, benzene, etc. It may be of practical importance in the treatment of wounds and chronic inflammations that further work be done on these lines. It appears that the lipoid membrane of cells, by taking up such substances as those mentioned, becomes more flexible, or, in other words, the surface tension is decreased and consequently amœboid movement is facilitated. From experiments made there is no doubt that phagocytosis is considerably accelerated in this manner. For instance, chloroform is seen to increase phagocytosis even in a dilution of one to five million.

Among other papers of interest to medical men may be mentioned one by Miss Smith-Rossie on "How Germany tries to abolish poverty and crime." A good description was given of the wide scope and power possessed by the body known as the *Armenpflege*, which exercised a restraining control over the home and did a great deal to prevent poverty, besides protecting illegitimate children.

